# Supplementary material for: HEMA-Lysine-Based Cryogels for Highly Selective Heparin Neutralization
Source: Int J Mol Sci. 2024 Jun 13;25(12):6503. doi: 10.3390/ijms25126503 (PMC11203617; doi:10.3390/ijms25126503)

Supplemental material

General procedure for ^{1}H-NMR titrations for pHEMA-lys – UFH or LMWH complexation.

Dry cryogel samples (10 mg) are chopped and inserted into an NMR tube containing 600 µL of D_2O and 0.5 µL of *tert*-butyl alcohol used as a reference for the calculation of NMR areas. Small aliquots of heparin solution at a concentration of 20 mg/ml are progressively added and the NMR tube is vortexed for a couple of minutes, then the area of the ^{1}H-NMR peak at δ = 1.95 characteristic of free heparin is monitored with respect to the area of the t-butanol peak at δ = 1.16.

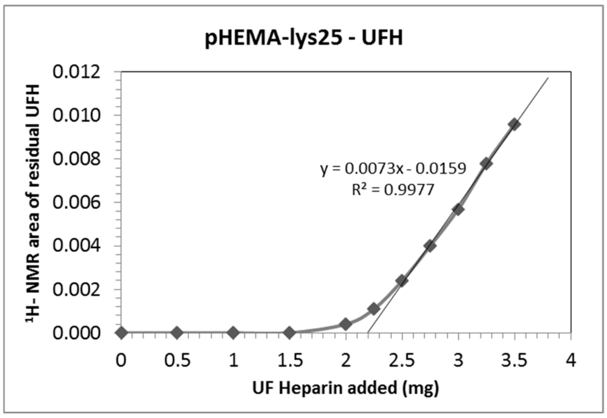

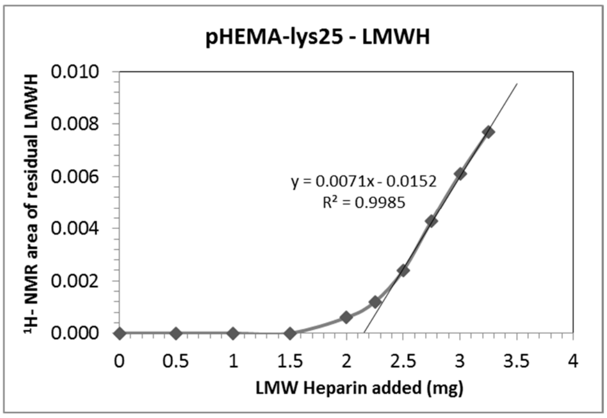

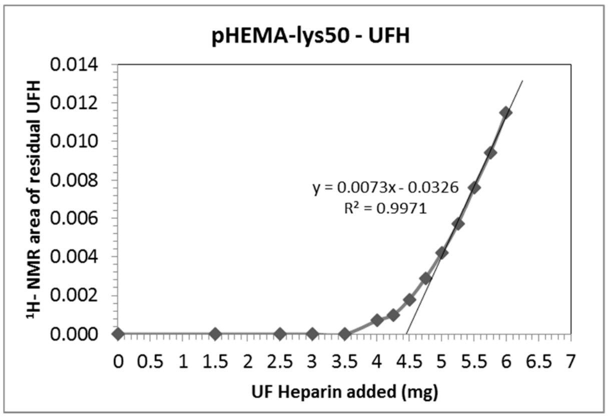

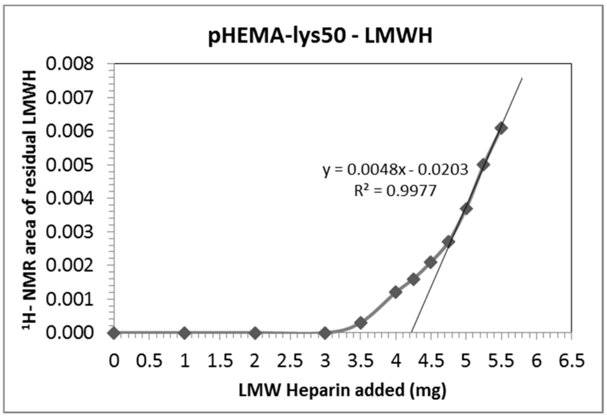

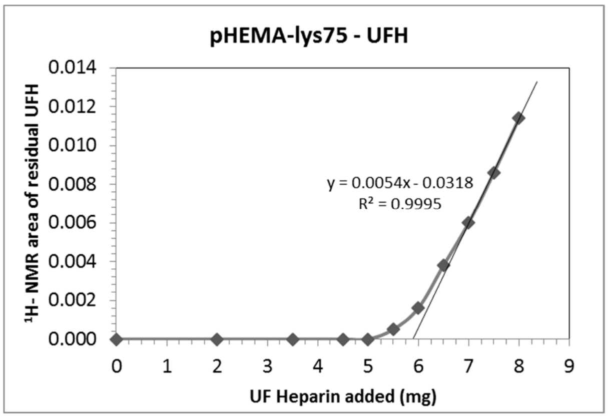

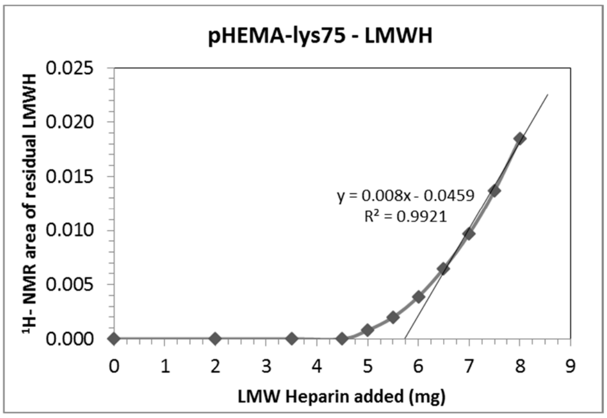

Supplement: Supplementary file 1 [file ijms-25-06503-s001.zip › ijms-3039883-supplementary.pdf]
